# Supplementary material for: Development and validation of a risk prediction algorithm for high-risk populations combining genetic and conventional risk factors of cardiovascular disease
Source: PLoS One. 2025 Oct 21;20(10):e0335064. doi: 10.1371/journal.pone.0335064 (PMC12539690; doi:10.1371/journal.pone.0335064)
Supplement: S1 Table — (PDF) [file pone.0335064.s001.pdf]

Table S1. Endpoint definitions

**Fatal cardiovascular disease**	
Hypertensive disease	I10–16
Ischemic heart disease	I20–25
Arrhythmias, heart failure	I46–52
Cerebrovascular disease	I60–69
Atherosclerosis/AAA	I70–73
Sudden death and death within 24h of symptom onset	R96.0–96.1
Excluding	
Myocarditis, unspecified	I51.4
Subarachnoid haemorrhage	I60
Subdural haemorrhage	I62
Cerebral aneurysm	I67.1
Cerebral arteritis	I68.2
Moyamoya	I67.5
**Non-fatal cardiovascular disease**	
Non-fatal ischemic heart disease	I21–25
Non-fatal stroke	I60–69
Excluding	
Subarachnoid hemorrhage	I60
Subdural hemorrhage	I62
Cerebral aneurysm	I67.1
Cerebral arteritis	I68.2
Moyamoya	I67.5
