# Supplementary material for: Development and validation of a risk prediction algorithm for high-risk populations combining genetic and conventional risk factors of cardiovascular disease
Source: PLoS One. 2025 Oct 21;20(10):e0335064. doi: 10.1371/journal.pone.0335064 (PMC12539690; doi:10.1371/journal.pone.0335064)
Supplement: S1 Text — (PDF) [file pone.0335064.s002.pdf]

Text S1. Management of predictors

All continuous predictors were centered and scaled before the analysis: age at 45 and by 5 years, BMI at 25 and by 5 kg/m2, SBP at 125 and by 20 mmHg, total cholesterol at 5 and by 1 mmol/L, HDL cholesterol at 1.5 and by 0.5 mmol/L. For BMI, values below 22 kg/m^2 or above 40 kg/m^2 were truncated at 22 and 40, respectively. For total cholesterol, values below 3 mmol/L or above 9 mmol/L were truncated at 3 and 9, respectively. Analogously, HDL cholesterol values were truncated at 0.7 mmol/L and 2.5 mmol/L. PRS for CAD was standardized to zero–mean and unit-variance.
