# Supplementary material for: Development and validation of a risk prediction algorithm for high-risk populations combining genetic and conventional risk factors of cardiovascular disease
Source: PLoS One. 2025 Oct 21;20(10):e0335064. doi: 10.1371/journal.pone.0335064 (PMC12539690; doi:10.1371/journal.pone.0335064)
Supplement: S2 Table — The models are derived in the full data of the earlier and later cohort using sex-specific and sex-stratified analysis for age groups 25–59 and 60 + , respectively. (PDF) [file pone.0335064.s003.pdf]

Table S2. Hazard ratios and p-values from CVD risk models with and without the PRS. The models are derived in the full data of the earlier and later cohort using sex-specific and sex-stratified analysis for age groups 25–59 and 60+, respectively.

Recruited 2002–2017						
	25–59				60+	
	Men		Women		Sex-stratified	
N CV events	8 554	1 196	19 365	1 638	4 635	2 176
	HR (95% CI)	P	HR (95% CI)	P	HR (95% CI)	P
Variable	Models without PRS					
Current smoking	1.59 (1.42–1.79)	<0.0001	1.41 (1.26–1.56)	<0.0001	1.48 (1.31–1.67)	<0.0001
SBP (per 20 mmHg)	1.26 (1.17–1.36)	<0.0001	1.26 (1.19–1.34)	<0.0001	1.06 (1.01–1.11)	0.013
BMI (per 5 kg/m2)	1.05 (0.97–1.13)	0.23	1.06 (1.00–1.12)	0.044	1.03 (0.98–1.09)	0.24
Total cholesterol (per 1 mmol/L)	1.09 (1.03–1.14)	0.0016	1.19 (1.13–1.24)	<0.0001	1.00 (0.96–1.04)	0.89
HDL cholesterol (per 0.5 mmol/L)	0.84 (0.77–0.93)	0.00036	0.80 (0.74–0.86)	<0.0001	0.89 (0.84–0.96)	0.0014
Variable	Models with PRS					
Current smoking	1.55 (1.38–1.74)	<0.0001	1.40 (1.26–1.56)	<0.0001	1.47 (1.30–1.66)	<0.0001
SBP (per 20 mmHg)	1.21 (1.13–1.31)	<0.0001	1.24 (1.17–1.32)	<0.0001	1.05 (1.00–1.11)	0.034
BMI (per 5 kg/m2)	1.05 (0.98–1.13)	0.19	1.06 (1.00–1.12)	0.049	1.03 (0.98–1.08)	0.27
Total cholesterol (per 1 mmol/L)	1.05 (1.00–1.11)	0.051	1.16 (1.11–1.22)	<0.0001	0.99 (0.95–1.03)	0.70
HDL cholesterol (per 0.5 mmol/L)	0.89 (0.81–0.97)	0.012	0.82 (0.76–0.89)	<0.0001	0.91 (0.85–0.97)	0.0059
PRS (per 1 SD)	1.35 (1.27–1.43)	<0.0001	1.17 (1.11–1.23)	<0.0001	1.10 (1.06–1.15)	<0.0001
Recruited 2018-2022						
	Men		Women		Sex-stratified	
N CV events	29 839	515	54 033	418	11 783	950
	HR (95% CI)	P	HR (95% CI)	P	HR (95% CI)	P
Variable	Models without PRS					
Current smoking	1.51 (1.25–1.82)	<0.0001	1.38 (1.09–1.74)	0.0066	1.65 (1.39–1.97)	<0.0001
SBP (per 20 mmHg)	1.14 (1.01–1.28)	0.029	1.20 (1.06–1.36)	0.0031	1.03 (0.95–1.11)	0.47
BMI (per 5 kg/m2)	1.09 (0.97–1.22)	0.16	1.05 (0.93–1.17)	0.43	1.04 (0.96–1.13)	0.31
Total cholesterol (per 1 mmol/L)	1.26 (1.15–1.37)	<0.0001	1.28 (1.16–1.41)	<0.0001	1.10 (1.03–1.18)	0.0043
HDL cholesterol (per 0.5 mmol/L)	0.69 (0.59–0.82)	<0.0001	0.71 (0.61–0.83)	<0.0001	0.79 (0.71–0.88)	<0.0001
	Models with PRS					
Current smoking	1.48 (1.23–1.79)	<0.0001	1.36 (1.07–1.71)	0.010	1.62 (1.36–1.93)	<0.0001
SBP (per 20 mmHg)	1.11 (0.99–1.25)	0.084	1.18 (1.04–1.33)	0.0077	1.01 (0.94–1.09)	0.72
BMI (per 5 kg/m2)	1.07 (0.95–1.20)	0.25	1.04 (0.93–1.17)	0.47	1.04 (0.96–1.13)	0.35
Total cholesterol (per 1 mmol/L)	1.21 (1.10–1.32)	<0.0001	1.24 (1.12–1.36)	<0.0001	1.08 (1.01–1.15)	0.021
HDL cholesterol (per 0.5 mmol/L)	0.73 (0.61–0.86)	0.00026	0.74 (0.63–0.87)	0.00017	0.82 (0.74–0.91)	0.00028
PRS (per 1 SD)	1.36 (1.24–1.49)	<0.0001	1.24 (1.12–1.37)	<0.0001	1.28 (1.20–1.37)	<0.0001
