# Supplementary material for: Development and validation of a risk prediction algorithm for high-risk populations combining genetic and conventional risk factors of cardiovascular disease
Source: PLoS One. 2025 Oct 21;20(10):e0335064. doi: 10.1371/journal.pone.0335064 (PMC12539690; doi:10.1371/journal.pone.0335064)
Supplement: S3 Table — The models are derived and the C-indices are calculated using the full data from the earlier and later cohort, with sex-specific and sex-stratified analyses for age groups 25–59 and 60 + , respectively. (PDF) [file pone.0335064.s004.pdf]

Table S3. Model discrimination in age and recruitment groups. The models are derived and the C-indices are calculated using the full data from the earlier and later cohort, with sex-specific and sex-stratified analyses for age groups 25–59 and 60+, respectively.

	Recruited 2002–2017			Recruited 2018–2022		
	25–59		60+	25–59		60+
	Men	Women	Sex-stratified	Men	Women	Sex-stratified
C-index of conventional model	0.6029	0.6077	0.5438	0.6012	0.6146	0.5690
C-index of conventional model with PRS	0.6311	0.6124	0.5515	0.6258	0.6150	0.5997
Difference in C-index	0.0282 (P<0.0001)	0.0047 (P=0.064)	0.0077 (P=0.04)	0.0246 (P=0.003)	0.0004 (P=0.48)	0.0307 (P=0.00048)
