# Supplementary material for: Development and validation of a risk prediction algorithm for high-risk populations combining genetic and conventional risk factors of cardiovascular disease
Source: PLoS One. 2025 Oct 21;20(10):e0335064. doi: 10.1371/journal.pone.0335064 (PMC12539690; doi:10.1371/journal.pone.0335064)
Supplement: S4 Table — The conventional model with and without PRS are compared separately in 25–59 and 60 + age group. (PDF) [file pone.0335064.s006.pdf]

Table S4. Categorical reclassification of 5-year CVD risk. The conventional model with and without PRS are compared separately in 25–59 and 60+ age group.

Age group 25–59									
		Conventional model with PRS				Reclassified			
	Conventional model	**<1.25%**	**1.25–5%**	**>5%**	Total	Lower	Higher	Net	NRI
CV event absent	**<1.25%**	47308	1314	0	48622	2244 (3.1%)	2014 (2.8%)	0.3%	3.0% (P=0.00102)
	**1.25–5%**	1589	17074	700	19363				
	**>5%**	0	655	4218	4873				
	Total	48897	19043	4918	7285				
CV event present		**<1.25%**	**1.25–5%**	**>5%**	Total	40 (3.5%)	71 (6.2%)	2.7%	
	**<1.25%**	206	28	0	234				
	**1.25–5%**	14	451	43	508				
	**>5%**	0	26	386	412				
	Total	220	505	429	1154				
Age group 60+									
		Conventional model with PRS				Reclassified			
	Conventional model	**<5%**	**5–10%**	**>10%**	Total	Lower	Higher	Net	NRI
CV event absent	**<5%**	2682	336	0	3018	752 (8.0%)	627 (6.7%)	1.3%	3.1% (P= 0.00185)
	**5–10%**	418	1965	291	2674				
	**>10%**	1	333	3363	3697				
	Total	3101	2634	3654	9389				
CV event present		**<5%**	**5–10%**	**>10%**	Total	60 (4.5%)	83 (6.3%)	1.7%	
	**<5%**	118	40	0	158				
	**5–10%**	31	190	43	264				
	**>10%**	0	29	868	897				
	Total	149	259	911	1319				
