# Supplementary material for: Development and validation of a risk prediction algorithm for high-risk populations combining genetic and conventional risk factors of cardiovascular disease
Source: PLoS One. 2025 Oct 21;20(10):e0335064. doi: 10.1371/journal.pone.0335064 (PMC12539690; doi:10.1371/journal.pone.0335064)
Supplement: S1 Fig — The calibration is assessed by sex, age group, and recruitment period. 10- and 5-year CVD risks are used for the earlier and the later cohort, respectively. (PDF) [file pone.0335064.s007.pdf]

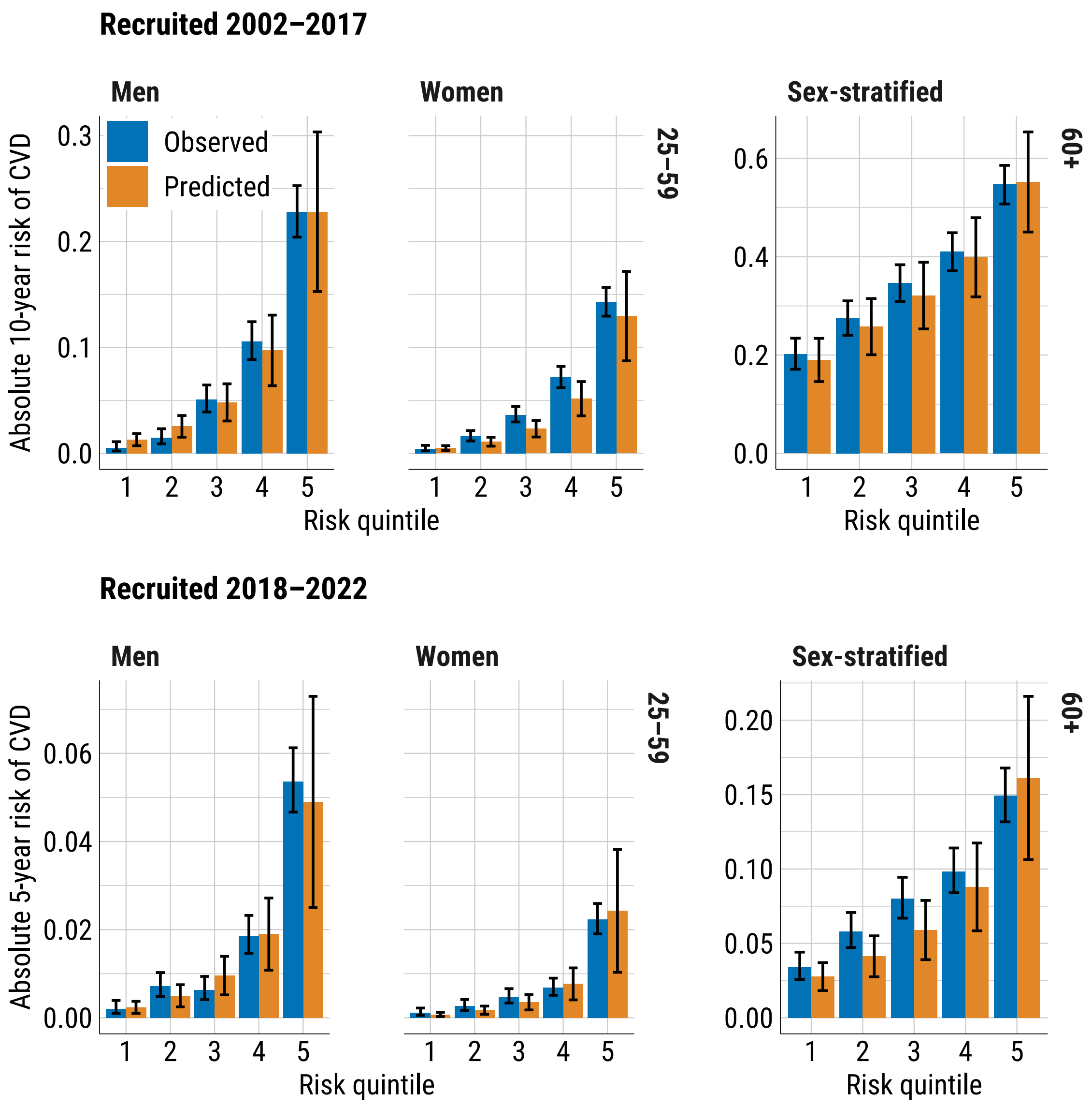

Fig S1. Calibration of absolute CVD risk in validation set. The calibration is assessed by sex, age group, and recruitment period. 10- and 5-year CVD risks are used for the earlier and the later cohort, respectively.
